# Computational gene network analysis reveals TNF-induced angiogenesis

**DOI:** 10.1186/1752-0509-6-S2-S12

**Published:** 2012-12-12

**Authors:** Kentaro Ogami, Rui Yamaguchi, Seiya Imoto, Yoshinori Tamada, Hiromitsu Araki, Cristin Print, Satoru Miyano

**Affiliations:** 1 Human Genome Center, The Institute of Medical Science, The University of Tokyo, 4-6-1 Shirokanedai, Minato-ku, Tokyo 108-8639 Japan; 2 Department of Computer Science, Graduate School of Information Science and Technology, The University of Tokyo, 7-3-1Hongo, Bunkyo-ku, Tokyo 113-0033 Japan; 3 Department of Molecular Medicine and Pathology, School of Medical Sciences, Faculty of Medical and Health Sciences, University of Auckland, Private Bag 92019, Auckland 1142, New Zealand

## Abstract

**Background:**

TNF (Tumor Necrosis Factor-α) induces HUVEC (Human Umbilical Vein Endothelial Cells) to proliferate and form new blood vessels. This TNF-induced angiogenesis plays a key role in cancer and rheumatic disease. However, the molecular system that underlies TNF-induced angiogenesis is largely unknown.

**Methods:**

We analyzed the gene expression changes stimulated by TNF in HUVEC over a time course using microarrays to reveal the molecular system underlying TNF-induced angiogenesis. Traditional *k*-means clustering analysis was performed to identify informative temporal gene expression patterns buried in the time course data. Functional enrichment analysis using DAVID was then performed for each cluster. The genes that belonged to informative clusters were then used as the input for gene network analysis using a Bayesian network and nonparametric regression method. Based on this TNF-induced gene network, we searched for sub-networks related to angiogenesis by integrating existing biological knowledge.

**Results:**

*k*-means clustering of the TNF stimulated time course microarray gene expression data, followed by functional enrichment analysis identified three biologically informative clusters related to apoptosis, cellular proliferation and angiogenesis. These three clusters included 648 genes in total, which were used to estimate dynamic Bayesian networks. Based on the estimated TNF-induced gene networks, we hypothesized that a sub-network including IL6 and IL8 inhibits apoptosis and promotes TNF-induced angiogenesis. More particularly, IL6 promotes TNF-induced angiogenesis by inducing NF-κB and IL8, which are strong cell growth factors.

**Conclusions:**

Computational gene network analysis revealed a novel molecular system that may play an important role in the TNF-induced angiogenesis seen in cancer and rheumatic disease. This analysis suggests that Bayesian network analysis linked to functional annotation may be a powerful tool to provide insight into disease.

## Background

Continuous TNF stimulation promotes proliferation and new blood vessel formation by HUVEC - the process of TNF-induced angiogenesis [1,2], which plays a role in the pathogenesis of solid tumours, multiple myeloma [3-5] and rheumatoid arthritis [1,2,6]. However, the molecular system underlying TNF-induced angiogenesis is not well understood [7]. Better understanding of this system may lead to new biomarkers an anti-cancer drugs.

## Methods

We analysed a DNA microarray data set (CodeLink™ Human Uniset I 20K) in which gene expression had been measured in HUVEC over eight time points of TNF stimulation (0, 1, 1.5, 2, 3, 4, 5 and 6 hours) in triplicate (GSE27870). Here, time 0 means the time point when exposure to TNF was started. The 8 time points × 3 replicate data set was normalized using cyclic Loess. Before cluster analysis below, we removed those genes that had average expression values across all time points ≤ 5 or missing values at any time point, leaving 3,673 genes. The expression data for each gene was then standardised so that mean = 0 and variance = 1.

We performed clustering using a *k*-means algorithm with Peason's correlation coefficient as the similarity measure between genes to identify sets of genes that have similar temporal gene expression patterns in HUVEC following TNF stimulation. We should note that other clustering techniques with different similarity metrics could also be used. For example with this time course data we could have used a similarity metric that incorporated a time lag between the compared expression profiles, however since the dataset used contained only eight time points we used non-time lagged statistic in order to achieve as stable a result as possible.

During the *k*-means clustering the number of clusters was set to 15. Although the number of clusters chosen stongly influences the output of *k*-means clustering algorithms and is usually statistically optimised, clustering is not a final goal of our analysis in this case. Therfore, a single relatively large value of *k *was chosen in order to obtain tight clusters that have closely correlated and possibly complex co-expression patters.

Then, to identify biologically informative clusters, we used functional enrichment analysis by DAVID (The Database for Annotation, Visualization and Integrated Discovery) [8]. The functional enrichment analysis DAVID was performed to identify genes in the same cluster enriched for particular biological functions. After finding biologically informative clusters, we needed to elucidate the putative directional relationships between the genes in these clusters. For this purpose, we estimated a probabilistic network of relationships between these genes using a Bayesian network estimation program, SiGN-BN [9,10], implemented on the supercomputer system at Human Genome Center, The Institute of Medical Science, The University of Tokyo [11]. The estimated gene network was analyzed using Cell Illustrator [12], a gene network analysis platform.

## Results

### Clustering and functional enrichment of clusters

We performed *k*-means clustering with *k *= 15 and evaluated the functional enrichment of genes in each cluster using DAVID. Of the 15 clusters, we found that three had significant enrichment of cell proliferation, apoptosis and angiogenesis annotations. The results of functional enrichment analysis using DAVID are summarized in Table 1. These three clusters included a total of 648 genes. The profiles of these genes in TNF-stimulated HUVEC were used to estimated a Baayesian gene regulatory network.

**Table 1:** Results of functional enrichment analysis for three clusters.

Category	Term	#genes	p-value
Cluster A			
GOTERM_BP_FAT	GO:0008283: cell proliferation	13	0.0027
GOTERM_BP_FAT	GO:0001775: cell activation	10	0.0039
GOTERM_BP_FAT	GO:0006916: anti-apoptosis	8	0.0070
GOTERM_BP_FAT	GO:0040007: growth	7	0.0144
KEGG_PATHWAY	hsa05222: Small cell lung cancer	5	0.0442
GOTERM_BP_FAT	GO:0001568: blood vessel development	7	0.0497

Cluster B			
GOTERM_BP_FAT	GO:0007596: blood coagulation	5	0.00034
GOTERM_BP_FAT	GO:0043066: negative regulation of apoptosis	8	0.0056
GOTERM_BP_FAT	GO:0043069: negative regulation of programmed cell death	8	0.0060
GOTERM_BP_FAT	GO:0060548: negative regulation of cell death	8	0.0061
GOTERM_BP_FAT	GO:0045765: regulation of angiogenesis	4	0.0066
GOTERM_BP_FAT	GO:0006916: anti-apoptosis	6	0.0083
GOTERM_BP_FAT	GO:0008283: cell proliferation	8	0.0164
KEGG_PATHWAY	hsa05200: Pathways in cancer	8	0.0237
GOTERM_BP_FAT	GO:0016477: cell migration	6	0.0260
PIR_SUPERFAMILY	PIRSF001719: fos transforming protein	2	0.0334
GOTERM_BP_FAT	GO:0008284: positive regulation of cell proliferation	7	0.0396
INTERPRO	IPR000837: Fos transforming protein	2	0.0443

Cluster C			
GOTERM_BP_FAT	GO:0006916: anti-apoptosis	9	0.0001
GOTERM_BP_FAT	GO:0043066: negative regulation of apoptosis	11	0.0001
GOTERM_BP_FAT	GO:0043069: negative regulation of programmed cell death	11	0.0001
GOTERM_BP_FAT	GO:0060548: negative regulation of cell death	11	0.0001
INTERPRO	IPR011539: Rel homology	3	0.0014
INTERPRO	IPR000451: NF-kappa-B/Rel/dorsal	3	0.0014
GOTERM_BP_FAT	GO:0043123: positive regulation of I-kappaB kinase/NF-kappaB cascade	5	0.0035
GOTERM_BP_FAT	GO:0043122: regulation of I-kappaB kinase/NF-kappaB cascade	5	0.0050
BIOCARTA	h_nthiPathway: NFκB activation by Nontypeable Hemophilus influenzae	4	0.0066
GOTERM_BP_FAT	GO:0016477: cell migration	7	0.0087
GOTERM_BP_FAT	GO:0030155: regulation of cell adhesion	5	0.0117
KEGG_PATHWAY	hsa05222: Small cell lung cancer	5	0.0125
PIR_SUPERFAMILY	PIRSF037644: inhibitor of apoptosis protein with CARD domain	2	0.0129
GOTERM_CC_FAT	GO:0033256: I-kappaB/NF-kappaB complex	2	0.0233
KEGG_PATHWAY	hsa05200: Pathways in cancer	9	0.0234
GOTERM_BP_FAT	GO:0030334: regulation of cell migration	5	0.0235

Functional enrichment for each cluster was evaluated by using DAVID. The following three clusters have enrichment of cell proliferation, apoptosis and angiogenesis that are important function for interpreting the estimated TNF-induced gene networks.

### Gene network analysis for TNF-regulated genes

**Figure 1:**
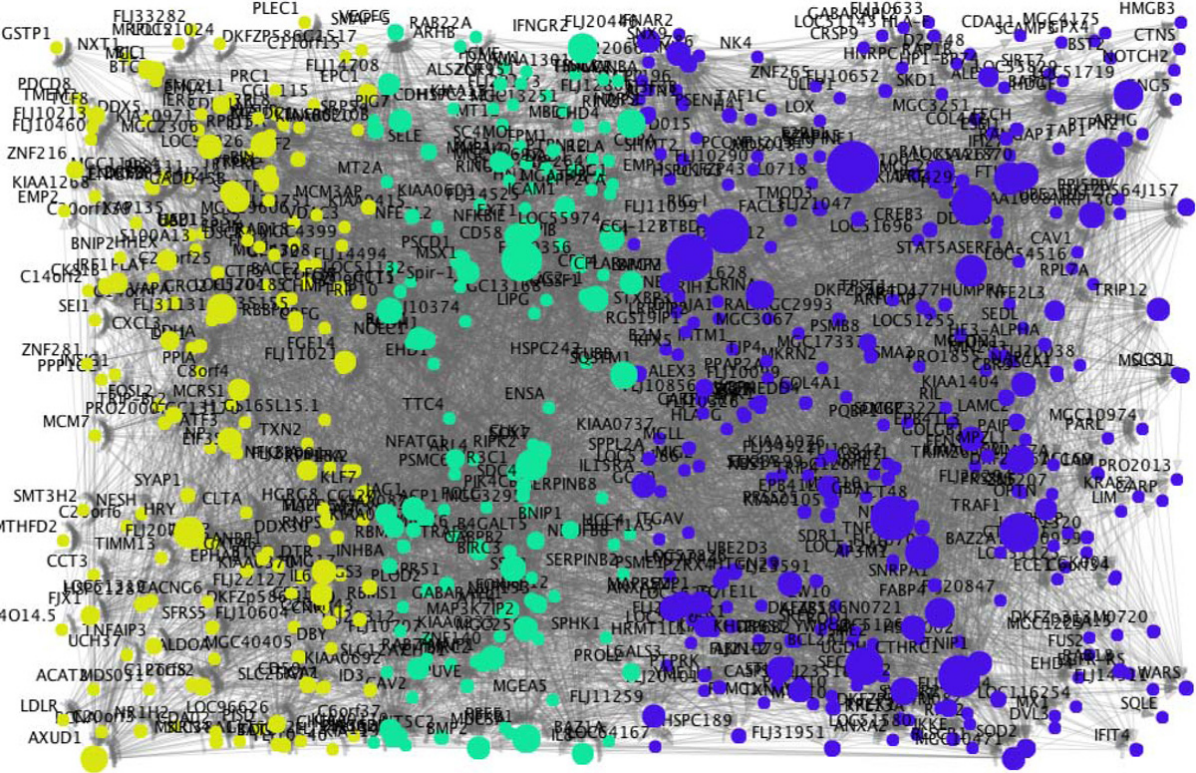
**Estimated gene network of 648 genes in three clusters that are functionally enriched for cellular proliferation, apoptosis and angiogenesis**. This gene network was estimated by SiGN-BN program containing 648 genes in three clusters that are functionally enriched for cellular proliferation, apoptosis and angiogenesis.The genes in clusters A, B and C are shown in yellow, green and blue colors, respectively.

Dynamic Bayesian networks were estimated using the computer program SiGN-BN (Figure 1), in which membership of the three clusters is distinguished by color. For more detail, the network file is also available (Additional file 1; csml format for Cell Illustrator). The size of each node is propotional to the number of its direct child genes, i.e., out-degree. Therefore, the genes shown as large nodes potentially function as hubs that are the candidate master regulators. Among the three clusters, angiogenesis genes are enriched in the cluster C. The expression levels of genes in the cluster C are induced in early time points, with the average expression profile of genes in this cluster show the peak at 1.5 hours after TNF treatment was initiated and then decreased monotonically. Interestingly, Interleukin-6 (IL6) is included in the cluster C. IL6 transduces proliferation-promoting signals and many proliferation-related genes are induced [13,14]. Therefore, we next focused on the sub-network related to IL6.

We have previously noted that especially important gene network hubs may have their direct chilren enriched for specific functional annotations [15], therefore we looked at the functional annotations of IL6 children. In the estimated network, IL6 has 48 direct child genes, including ten genes related to the Gene Ontology (GO) path for cellular proliferation (GO:0008283; BTG3, CDC37, CDKN1A, CKS1B, EMP2, IL8, INHBA, PPP1CC, PRC1, RASSF1) and 40 genes known to be NF-kB targets, with Transfac promoter motif V$NFKB_Q6_01 (BAT8, BTG3, C14orf2, C20orf3, CAV2, CCL20, CCT3, CDC37, CDKN1A, CKS1B, CSF2, DIA1, EMP2, EPHA2, FLJ10374, GABPB2, HN1, IER5, IL8, INHBA, LDLR, MGC4308, MRPL12, NDUFB8, NFATC1, NFE2L2, PMAIP1, PPP1CC, PRC1, RASSF1, RGS3, RING1, SDC4, SELE, SLC12A7, SS18, TIMM13, TNFRSF10B, USP12, YTHDF2).

**Figure 2:**
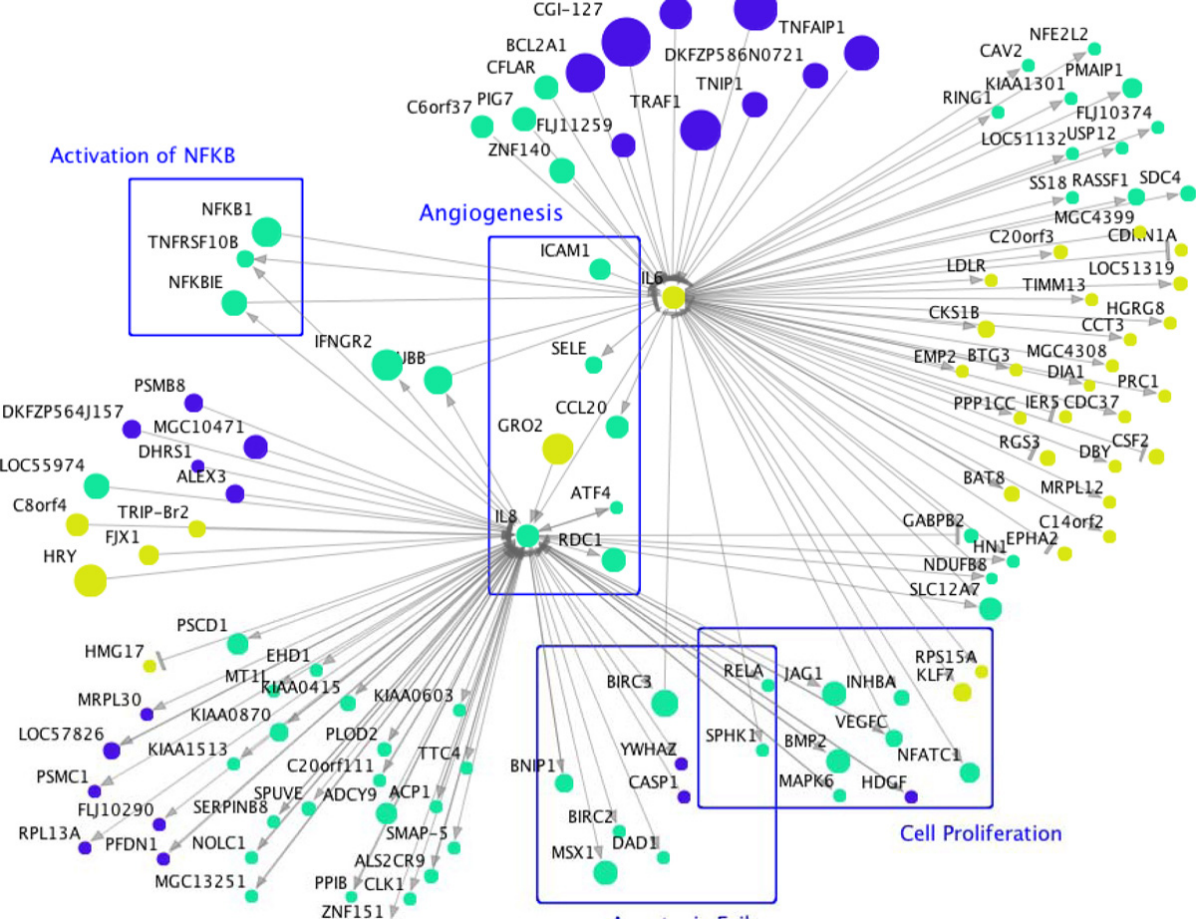
**IL6 and IL8 related gene network**. The direct parents and children of IL6 in the estimated gene network were extracted. The genes that are direct parents and children of IL8 that is a child gene of IL6 were also added.

NF-κB is a master transcription factor, which induces genes that control cell proliferation, cell survival, and immune response [16]. Although no genes encoding NF-κB family proteins are direct children of IL6, we have previously suggested that the NF-κB family do not appear in gene networks [17], since the activity of this family are regulated largely by post-transcriptional mechanisms. However, IL6 may not work alone in regulating NF-κB transcriptional activity. Its activity may be complimented by TNF-induced molecular pathways that converge on NF-κB from several directions, since 23 other hubs in the gene network also have ≥10 direct children that are known in the Transfac database to have NF-κB response elements in their promoters (CGI-127, KIAA0429, RIG-I, TAP1, CD58, BCL2A1, GRO2, TRAF1, STAT5A, HHEX, MGC10471, FLJ11259, MSX1, FLJ14708, BCL3, BIC, IRF1, BMP2, BIRC3, TNIP1, LOC51267, CFLAR, DKFZP586N0721). Interestingly, Interleukin-8 (IL8) is also a direct child gene of IL6 in the gene network. IL8 induces angiogenesis by strongly promoting HUVEC growth [7,18]. Two direct children of IL8 are also key to NF-κB family activity - RELA is an NF-κB family member and NFKBIE is a direct upstream regulator of NF-κB proteins. Based on these facts, we propose the hypothesis that IL6, along with cascade of other molecules, works as an initiator of TNF-induced angiogenesis to promote excess HUVEC growth by inducing NF-κB and IL8. The sub-network related to IL6 and IL8 is shown in Figure 2.

As for the estimated relationship between IL6 and IL8, it is known that IL6, TNF and IL1 combinatorially induce NF-κB and NF-IL6 (nuclear factor interleukin 6), which in turn induce IL8 [19]. Therefore, under TNF stimulus, our estimated edge from IL6 to IL8 is biologically tenable based on published experimental results.

The network also suggests a relationship of IL6 and IL8 to apoptosis. NFκB1, CFLAR, BCL2A1 STAT5A and TRAF1 are anti-apoptotic factors and are estimated to induce IL6, i.e., they are direct network parents of IL6. On the other hand, IL8 is known to inhibit endothelial cell apoptosis [20]. In the gene network, five genes (BIRC2, BIRC3, BNIP1, POGK, RELA) of the 50 genes that are direct children of IL8 are annotated with an anti-apoptotic function (GO:0043066). Therfore, the network suggests a complex TNF-indiced apoptosis-regulating pathway focussed around IL6 and IL8.

In addition, in the IL6-IL8 sub-network, IL8 and IL6 have eight and ten children, related to cellular proliferation (GO:0008283). The TNF-induced pathways proposed by this network analysis that lie downstream of IL6 and IL8 may synergise to promote TNF-angiogenesis, since it is known that the processes of proliferation and apoptosis are both required for angiogenesis [21].

## Discussion

Our computational gene network suggetsed that TNF-induced angiogenesis may be promoted by modulating both apoptosis and proliferation via IL6-IL8 sub-networks initiated by TNF. IL8 is a major angiogenesis factor, but the mechanism of IL8 induction is not fully understood [7]. Our anlalysis suggests that the roles played by IL6 and IL8 in regulating NF-kB activity, apoptosis and cellular proliferation during TNF-induced angiogenesis should receive further experimental study. This is cliniclaly relevant since the TNF-induced angiogenesis inhibitor thalidomide is used for treatment of cancers [3-5], and since the TNF-induced activation of NF-κB transcripional programs that occurs in breast cancer and cultured endothelial cells appear very similar [22].

## Conclusions

TNF-induced angiogenesis is an important factor in cancer and rheumatic disease [1,2,6]. Computational gene network analysis proposed new biologically tenable molecular pathways of TNF-induced angiogenesis. In particular, based on the information of the TNF-induced estimated gene network, IL6 induced NF-κB [7] and IL8 [19] appear to play an especially strong role in this process. This study suggests that the analysis of drug response time course gene expression data using gene network methods, along with functional annotation of the gene networks, has the potential to retrieve information about complex molecular systems, and that this method can be used to generatre hypotheses for testing in the laboratory.

## Competing interests

The authors declare that they have no competing interests.

## Authors' contributions

KO, RY and SI conceived and designed the analysis. KO performed the analysis. CP, HA and YT contributed data and analysis tools. SM supervised the research. KO wrote the paper. All authors read and approved the final manuscript carefully.
